# Effect of ethylenediaminetetraacetic, etidronic, and peracetic acids with different concentrations on the removal of *Enterococcus faecalis* biofilms from root canal walls: an *in vitro* study

**DOI:** 10.1007/s11274-026-04823-2

**Published:** 2026-03-06

**Authors:** Salev Zeyrek, Özgür İlke Ulusoy, Gülçin Akca, İlke Gaye Savur

**Affiliations:** 1 Department of Endodontics, Faculty of Dentistry, Gazi University, 82.Street, Ankara, Emek 06510 Turkey; 2 Department of Microbiology, Faculty of Dentistry, Gazi University, Ankara, Turkey

**Keywords:** Etidronic acid, E.faecalis, Biofilm, Peracetic acid, Root canal

## Abstract

E. faecalis is one of the most important microbiological factors responsible for the failures after root canal treatment. The knowledge about the efficacy of HEBP and PAA with different concentrations, when they are used alone or associated with NaOCl on E. faecalis biofilms is limited. In the present study, the efficacy of 17% ethylenediaminetetraacetic acid (EDTA), 9% and 18% etidronic acid (HEBP), and 1% and 2% peracetic acid (PAA), used alone or in combination with sodium hypochlorite (NaOCl), was evaluated on dentine discs experimentally infected with Enterococcus faecalis biofilms using a laser scanning confocal microscope. Irrigation of the samples using HEBP solutions in combination with NaOCl decreased the viability of E.faecalis more effectively compared with the sole use of 9% HEBP and 18% HEBP solutions. The use of two different concentrations of peracetic acid (1–2%) and etidronic acid (9–18%) resulted in similar biofilm elimination when these chelators were used alone or in combination with NaOCl. The sole use of 2% peracetic acid in the experimentally infected samples eliminated more biofilm than the use of 9% and 18% HEBP. Lower concentrations of peracetic acid and etidronic acid can be recommended to remove the E.faecalis biofilms from root canals to decrease the irrigation solutions’ potential harmful effects.

## Introduction

The primary goal of endodontic treatment is to eliminate the microorganisms from the root canal system, provide conditions that will not allow microbial proliferation, and obtain periradicular tissue healing (Siqueira and Roças 2008; Jhajharia et al. 2015). The microorganisms in the infected root canal system exist in planktonic or biofilm formation (Lin et al. 2013). Bacteria resistant to root canal treatment procedures are commonly located in the biofilm structures and firmly adhere to each other and root canal walls (Lin et al. 2013; Sonja et al. 2013). *E. faecalis*, a facultative anaerobic gram-positive coccus, is the most frequent resistant species isolated from root canal biofilms (Liu et al. 2010; Sedgley et al. 2005). This bacteria can survive after conventional chemomechanical procedures due to its adaptation capacity to hard environmental changes and invasion ability to dentinal tubules (Ricucci and Siqueira 2010; Parolia et al. 2021). Therefore, the *E. faecalis* biofilm model is generally used in studies that evaluate the different irrigation and disinfection protocols regarding their antibiofilm capacity (Kishen and Haapasalo 2010).

Irregular areas of the root canal system, such as lateral canals, apical ramifications, and isthmuses that are difficult to access with mechanical instrumentation, are convenient regions for bacterial biofilms (Nair et al. 2005). Therefore, the efficacy of irrigation and disinfection protocols is significant in eliminating biofilms from the root canal system. Sodium hypochlorite (NaOCl) is the most widely used irrigation solution in root canal treatment due to its superior antimicrobial effect and organic tissue dissolving ability (Mohammadi 2008). However, due to its limited capacity to remove inorganic compounds from the smear layer from root canal walls, it is recommended to use NaOCl with a decalcifying agent for successful irrigation (Boutsioukis and Ariaz-Moliz 2022). For this purpose, ethylenediaminetetraacetic acid (EDTA) is the most widely used chelating agent combined with NaOCl. However, EDTA has a limited ability to remove the smear layer from the apical third of the root canal, and it decreases the tissue-dissolving and antimicrobial effect of NaOCl in combined use (De-Deus et al. 2008; Nogo Zivanovic et al. 2019). For these reasons, different chelating agents that will not reduce the antibacterial and tissue-dissolving capacities of NaOCl have been investigated.

1-hydroxyethylidine-1, 1-bisphosphonate, also known as etidronic acid (HEBP), is a weak chelating agent. Etidronic acid is a bisphosphonate that does not have toxic effects on tissues and is used in personal cleaning products and swimming pools as a disinfectant due to its antimicrobial properties (Russell and Rogers 1999). In recent years, etidronic acid has been suggested as an alternative chelating agent to EDTA that shows minimal interaction with NaOCl (Arias-Moliz et al. 2016). This solution has also been recommended to be used together with NaOCl as a single irrigation solution (Villalta-Briones et al. 2021). This mixture (NaOCl + HEBP) was suggested to remove the smear layer efficiently and provide sufficient tissue-dissolving and antimicrobial ability in the root canal (Girard et al. 2005).

Peracetic acid (PAA) is another weak chelating agent with antimicrobial activity against spores, viruses and fungi (Lensing and Oei 1985). Acetic acid released from peracetic acid solution can form water-soluble compounds with calcium (Lottanti et al. 2009). It has previously been reported that peracetic acid has a similar smear layer removal ability to EDTA (Ulusoy et al. 2017).

Many methods are being used to examine the antimicrobial effects of the irrigation protocols on in vitro and ex vivo biofilms (Boutsioukis et al. 2022). Confocal laser scanning microscope (CLSM) is a novel technique assessing root canal bacteria’s viability, location, and distribution (Flach et al. 2016). There are research studies in the literature evaluating the effect of different irrigation protocols on the *E.faecalis* biofilms (Arias-Moliz et al. 2016; Flach et al. 2016). However, there is limited knowledge regarding the effect of concentrations of the irrigation solutions on their antimicrobial capacity (Briseño-Marroquín et al. 2022). In addition, there is no consensus about the influence of interactions between the irrigants on eliminating biofilms from the root canal system. This study aimed to determine the effects of 17% ethylenediaminetetraacetic acid (EDTA), 9% and 18% etidronic acid (HEBP), and 1% and 2% peracetic acid (PAA) used alone or in combination with NaOCl on *Enterococcus faecalis* biofilms by using a laser scanning confocal microscope. The null hypothesis tested was that there is no difference between the irrigation protocols regarding elimination of *E.faecalis* biofilms from root canal dentinal walls.

## Methodology

This study was approved by the institutional ethics committee (No: 21071282-050.99-). Freshly extracted human premolar teeth with a single root and one root canal were selected. Teeth with caries, cracks, fractures, calcified root canals, immature apices, root resorption, and previous endodontic treatment were excluded. The presence of a single canal was confirmed with periapical radiographs. One hundred and fifteen teeth meeting these criteria were stored in sterile water at 4℃ before the experiment.

### Preparation of dentine discs and generation of *E. faecalis* biofilms

**Fig. 1:**
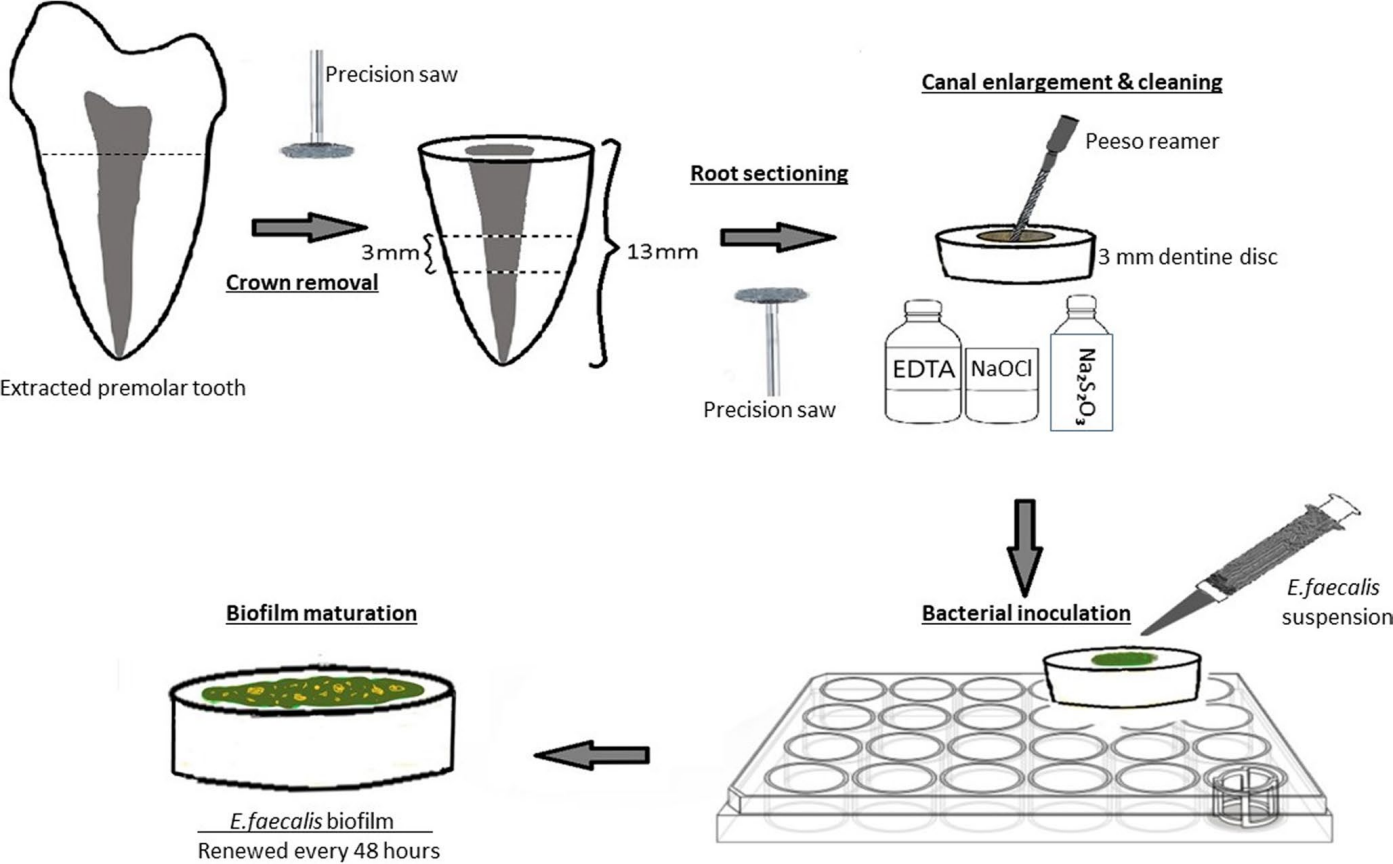
Schematic illustration summarizing the preparation of dentine discs and biofilm maturation

The residual tissues on the root surfaces of the teeth were cleaned using periodontal scalers. The teeth were separated from their crowns under water cooling, remaining with a root length of 13 mm. Each root was sectioned horizontally at the middle third to obtain one slice with a thickness of 3 mm using a precision saw (Isomet, Buehler, NY, USA) (Fig. 1). The thickness of each slice was confirmed using a digital caliper.

The root canal space of the dentine slices was enlarged to a size of 1 mm using a #2 peeso reamer (Mani, Tochigi, Japan). The dentine discs were irrigated using 17% EDTA solution (Wizard, Rehber Chemistry, Turkey) for 1 min to remove the smear layer. They were then washed with 2.25% sodium hypochlorite (NaOCl) (Wizard). The action of NaOCl was neutralized with 5% sodium thiosulfate. The specimens were sterilized using ethylene oxide. *Enterococcus faecalis* ATCC #29,212 standard strain was produced by incubation at 37℃ for 24 h in petri dishes containing tryptic soy agar (Merck, Darmstadt, Germany) (TSA). Purity controls were performed on the produced colonies. The produced cell suspension was then adjusted in 100 × 15 mm sterile glass tubes containing 10 mL tryptic soy broth (TSB) (Merck) to match the turbidity of 1.5 × 10^8^ colony forming unit (CFU/mL) equivalent to 0.5 McFarland standards. The determined number of sterile dentine discs in each group was placed on sterile polystyrene 24-well cell culture plates for biofilm development. Sterile pipettes transferred 100 µL of the *E. faecalis* suspension into the dentine discs. Then, 2 mL of TSB growth culture was added to cover the whole surface of the prepared dentine disc. One hundred µL of 5% sucrose concentration was added to the dentine discs. The dentine specimens were incubated at 37 °C in an aerobic atmosphere for seven days. After 24 h, the samples were checked for bacterial growth. The specimens were replenished with *E. faecalis* biofilm every 48 h to manage biofilm formation and supply nutrients to the bacteria ensuring their survival (Fig. 1).

### Treatment of the infected dentine discs with different irrigation protocols

One hundred fifteen infected dentine samples were randomly divided into 11 experimental (*n* = 10) and one control group (*n* = 5) according to the irrigation regimen:

#### **Group 1** [6 mL 2.5% sodium hypochlorite (NaOCl)]

2.5% NaOCl was obtained by mixing 5.25% NaOCl (Wizard, Rehber Chemistry, Istanbul, Turkey) with water in equal proportions. The solution was applied manually using a disposable dental syringe (Ultradent Products, South Jordan, UT, USA) and a 27-G needle (NaviTip) for 2 min without any activation.

#### **Group 2** [6 mL 17% ethylene diamine tetraacetic acid (EDTA)]

17% EDTA (Wizard, Rehber Chemistry, Istanbul, Turkey) was purchased from a commercial source at the indicated concentrations. The solution was applied manually for 2 min as described above.

#### **Group 3** [6 mL 1% peracetic acid (PAA)]

A 36–40% peracetic acid solution (Code: 433241) supplied from a company (SigmaAldrich, St Louis, MO) was diluted with non-ionized water to obtain a solution with a weight/volume ratio of 1%. This solution was stored in the refrigerator at + 4 °C and warmed to room temperature before use. The solution was applied manually for 2 min as described above.

#### **Group 4** [6 mL 2% peracetic acid (PAA)]

A 36–40% peracetic acid solution (Code: 433241) (SigmaAldrich, St Louis, MO) was diluted with non-ionized water to obtain a solution with a weight/volume ratio of 2%. This solution was stored in the refrigerator at + 4 °C and warmed to room temperature before use. The solution was applied manually for 2 min as described above.

#### **Group 5** [6 mL 9% etidronic acid (HEBP)]

Aqueous 60% HEBP solution (Code: H6773) was obtained from a commercial market (SigmaAldrich, St Louis, MO). It was mixed with ultrapure water with a weight/volume ratio of 9% and stored at room temperature in a glass bottle until use. The solution was applied manually for 2 min as described above.

#### **Group 6** [6 mL 18% etidronic acid (HEBP)]

Aqueous 60% HEBP solution (Code: H6773) was obtained from a commercial market (SigmaAldrich, St Louis, MO). It was mixed with ultrapure water with a weight/volume ratio of 18% and stored at room temperature in a glass bottle until use. The solution was applied manually for 2 min as described above.

#### **Group 7** [3 mL 2.5% NaOCl- 3 mL 17% EDTA]

3 mL 2.5% NaOCl solution was applied manually using a disposable dental syringe (Ultradent Products, South Jordan, UT, USA) and 27-G needle (NaviTip) for 1 min without any activation. Then, 3 mL of 17% EDTA was applied manually as stated above for 1 min.

#### **Group 8** [3 mL 2.5% NaOCl- 3 mL 1% PAA]

3 mL 2.5% NaOCl solution was applied manually for 1 min as stated above. Then, 3 mL 1% PAA was applied manually for 1 min.

#### **Group 9** [3 mL 2.5% NaOCl- 3 mL 2% PAA]

3 mL 2.5% NaOCl solution was applied manually for 1 min as stated above. Then, 3 mL 2% PAA was applied for 1 min.

#### **Group 10** [6 mL 2.5% NaOCl + 9% HEBP]

A 6 mL 2.5% NaOCl + 9% HEBP single irrigation solution was obtained by mixing 3 mL 5.25% NaOCl solution and 3 mL 18% HEBP solution. This solution was applied manually for 2 min as described above.

#### **Group 11** [6 mL 2.5% NaOCl + 18% HEBP]

A 6 mL 2.5% NaOCl + 18% HEBP single irrigation solution was obtained by mixing 3 mL 5.25% NaOCl solution and 3 mL 36% HEBP solution. This solution was applied manually for 2 min as described above.

#### **Group 12** [Saline (control)]

6 mL saline solution was applied for 2 min as described above.

All the solutions were delivered with the application rate of 1 min/3mL. After the application period, 6 mL of water was added to neutralize the solution.

### Investigation of bacterial biofilms with confocal laser scanning microscope (CLSM)

After the irrigation regimen, all the experimentally infected dentine discs were stained with Live/Dead BacLightTM Bacterial Molecular Kit L7012 (Molecular Probes, Invitrogen, Carlsbad, CA, USA), which includes Syto-9 and propidium iodide (PI). Styo-9 is a green fluorescent stain and targets the intact membranes of the live bacteria. Propidium iodide is a red fluorescent stain and marks damaged membranes of the dead bacteria. According to the manufacturer’s guidelines, 9 µL dye was added to 3 mL distilled water. The mixture was vortexed for 2 min to obtain homogeneity. Following staining the samples using a 200 µL dye mixture for 15 min, each sample was washed with saline solution. A specialist operator examined each sample under a confocal laser scanning microscope *(*LSM 510 META, Zeiss, Jena, Germany) at 20x magnification. The recorded 2D images were converted to 3D images using Imaris 9.2.1 software (Bitplane, Oxford Instrument Company, Concord, MA, USA). The biological volumes of the dead and live bacteria were calculated and recorded. Then, the percentage of the dead bacterial biovolume was calculated using the data.

### Statistical analysis

The data was statistically analyzed using SPSS (Statistical Package for Social Sciences) version 15. Data were presented as means and standard deviations. The suitability of quantitative variables to normal distribution was investigated by the Shapiro-Wilk test. As the data was normally distributed, the Tukey multiple comparison test with one-way ANOVA was used to determine the significant differences between the groups. A p-value less than 0.05 was considered to be statistically significant.

## Results

**Fig. 2:**
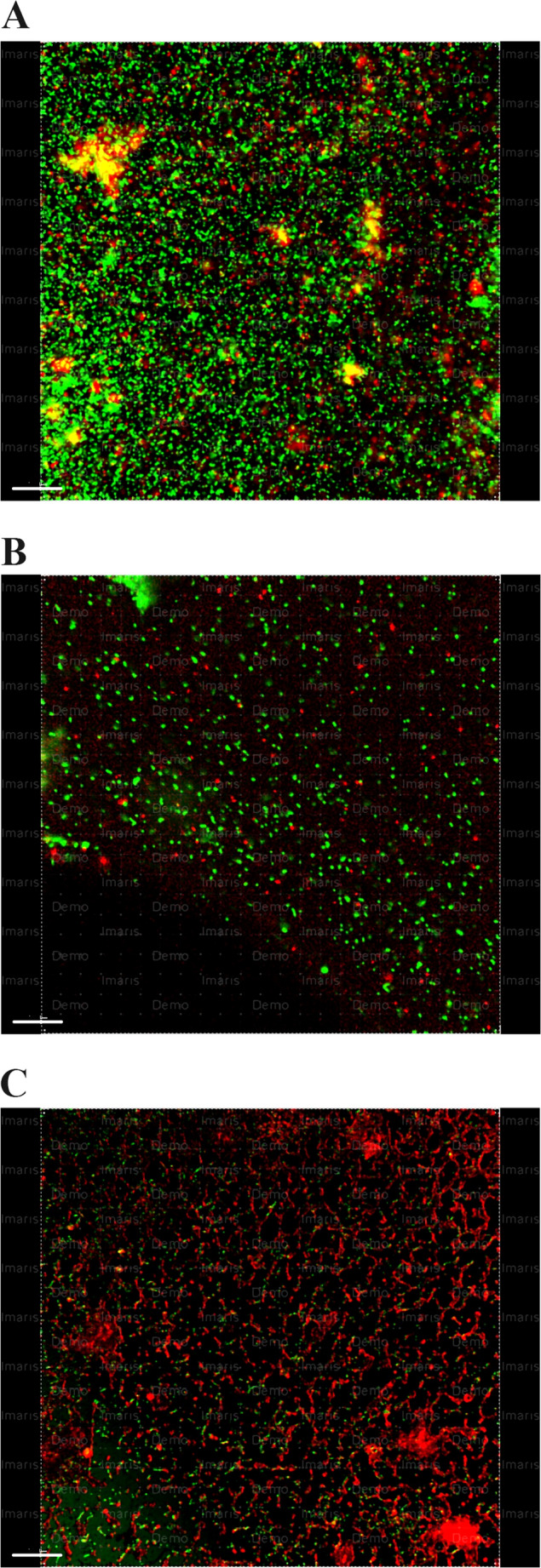
Representative CLSM photos showing dead and live bacteria within dentine discs after irrigation with (**a**) Saline, (**b**) 17% EDTA, (**c**) 1% PAA. Green color: live cells, red color: dead cells.

The mean and standard deviation values ​​of the dead bacteria percentage are shown in Table 1. The use of 2.5% NaOCl-2% PAA resulted in the highest values of dead cell percentage. However, the values ​​obtained from this group are not statistically different from those of the other groups. The percentage of the dead bacterial cells in the samples irrigated using 17% EDTA and saline were significantly lower compared to the samples irrigated with other irrigation regimens (*p* < 0.001) (Fig. 2).

**Table 1:** Means, standard deviations, minimum and maximum values for the percentages of the dead cells after exposure to different irrigation regimens

Irrigation protocol	Mean±SD	Min-Max	Post-hoc
2.5% NaOCl	0.836±0.096	0.710–0.979	A
17% EDTA	0.190±0.096	0.075–0.367	C
1% PAA	0.729±0.107	0.554–0.873	A, B
2% PAA	0.828±0.138	0.625–0.977	A
9% HEBP	0.599±0.126	0.414–0.818	B
18% HEBP	0.616±0.135	0.461–0.815	B
2.5% NaOCl – 17% EDTA	0.738±0.089	0.560–0.905	A, B
2.5% NaOCl – 1% PAA	0.852±0.166	0.509–0.995	A
2.5% NaOCl – 2% PAA	0.883±0.100	0.693–1.000	A
2.5% NaOCl + 9% HEBP	0.862±0.129	0.540–0.955	A
2.5% NaOCl + 18% HEBP	0.879±0.093	0.686–0.971	A
Saline (Control)	0.057±0.042	0.024–0.124	C

*SD* standard deviation, *min-max* minimum-maximum, *NaOCl* sodium hypochlorite, *EDTA* ethylenediaminetetraacetic acid, *HEBP* etidronic acid, *PAA* peracetic acid

The different letters indicate statistically significant differences between the groups (*p* < 0.05)

**Fig. 3:**
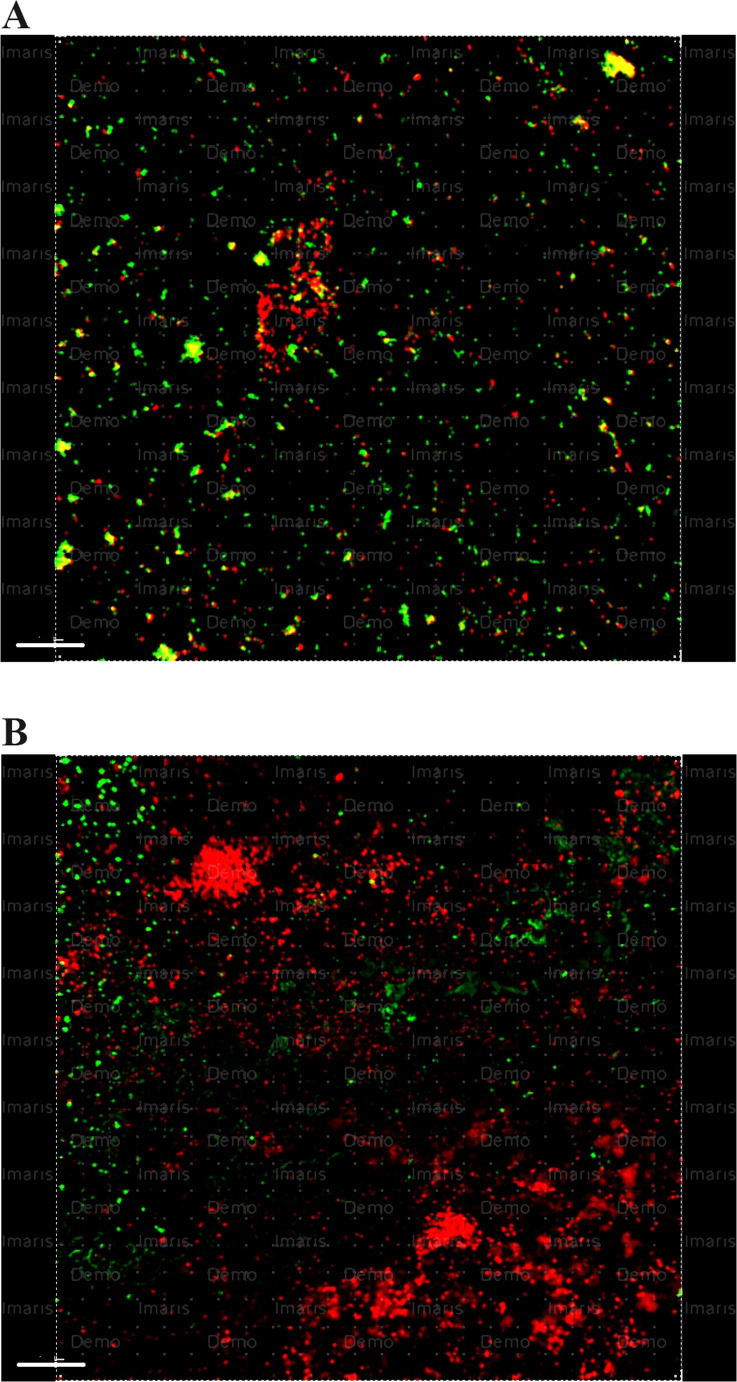
Representative CLSM photos showing dead and live bacteria within dentine discs after irrigation with (**a**) 9% HEBP, (**b**) 2.5% NaOCl + 9% HEBP. Green color: live cells, red color: dead cells

The percentage of dead bacteria observed in the samples irrigated using 9% HEBP and 18% HEBP solutions alone is lower compared to the samples irrigated using HEBP in combination with NaOCl (*p* < 0.001) (Fig. 3).

There was no statistically significant difference between the two concentrations of peracetic acid (1–2%) and etidronic acid (9–18%) regarding biofilm elimination, when these chelators were used alone or in combination with NaOCl (*p* > 0.001). However, the percentage of dead bacteria was lower in the samples irrigated with lower concentrations of PAA and HEBP, compared to the ones irrigated with higher concentrations.

**Fig. 4:**
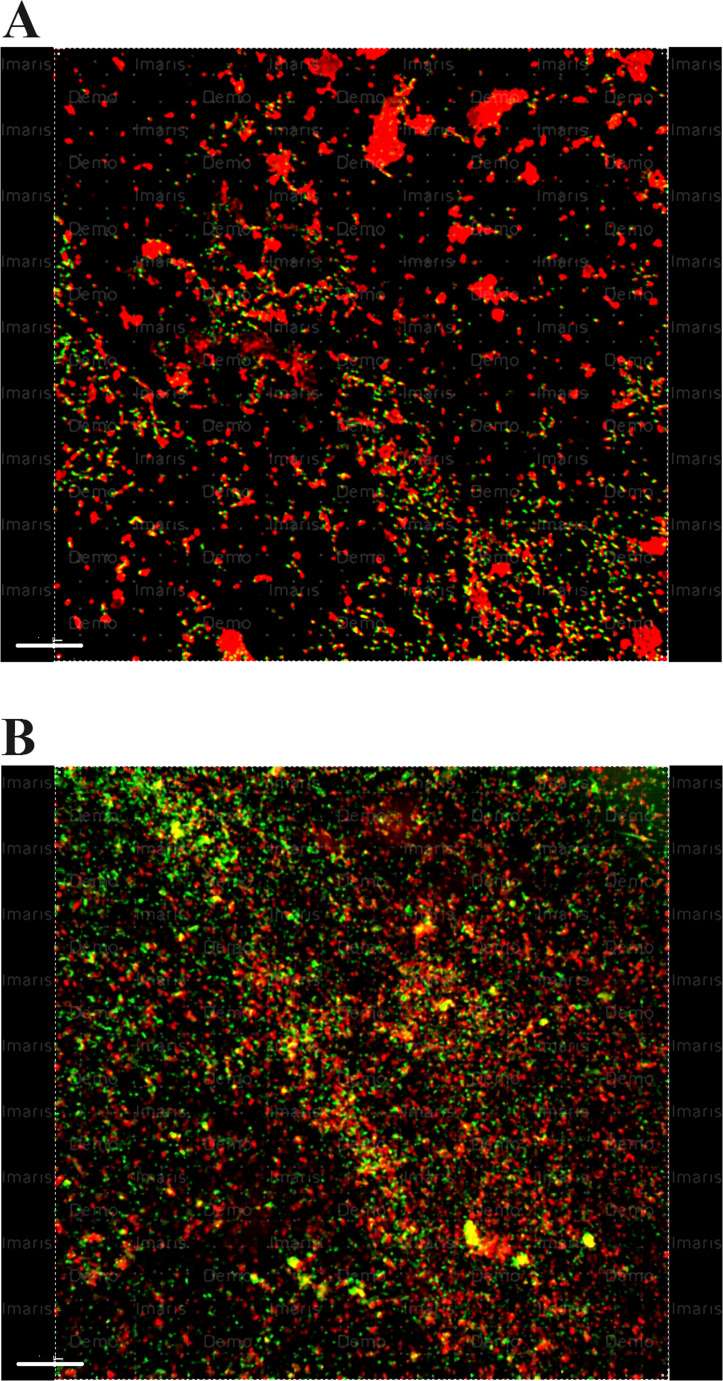
Representative CLSM photos showing dead and live bacteria within dentine discs after irrigation with (**a**) 2% PAA, (**b**) 18% HEBP. Green color: live cells, red color: dead cells

The use of 2% peracetic acid alone in the experimentally infected samples resulted in more biofilm elimination than the use of 9% and 18% HEBP alone (*p* < 0.001) (Fig. 4).

## Discussion

*E. faecalis* is one of the most important microbiological factors responsible for the failures after root canal treatment (Molander et al. 1998). There is little evidence in the literature regarding the efficacy of HEBP and PAA with different concentrations, when used alone or associated with NaOCl on *E. faecalis* biofilms (Briseño-Marroquín et al. 2022). In the present study, although the difference is not statistically significant, use of HEBP and PAA with higher concentrations eliminated higher amounts of *E. faecalis* biofilm, when these chelators were used alone or in combination with NaOCl. However, the antibacterial effect is not the only factor to determine the ideal concentration of the irrigation solution when using it in the root canal. It has previously been reported that undesirable effects of the irrigation solutions, such as toxicity, dentine erosion, and excessive decrease in dentin microhardness, are more frequently observed at higher concentrations (Kaya et al. 2011; Silva et al. 2012; Souza et al. 2021). Also, determining the ideal concentration of the irrigant regarding the smear layer removal capacity is another important factor that has to be considered. The lowest concentration that minimizes the potential harmful effects of the irrigation solution, when providing effective elimination of the smear layer and antibacterial capacity, should be preferred to be used in the root canal.

The use of 2% PAA provided significantly more effective biofilm elimination than 9% and 18% HEBP in the present research. Thus, the null hypotheses were rejected. This can be explained by the lower antibacterial effect of etidronic acid compared to peracetic acid (Tartari et al. 2018). However, when HEBP solution was used in combination with NaOCl, it showed similar antimicrobial effect with PAA used with NaOCl. This may be because HEBP does not react with NaOCl and does not limit its antibacterial effect (Arias-Moliz et al. 2014). The results of the present study support the findings of the previous ones, which have reported that the combined use of NaOCl and HEBP does not have an undesirable effect on the chemical properties of NaOCl (Tartari et al. 2015). In addition, the present results showed that the use of 9% and 18% HEBP has led to a slight increase in the antibacterial capacity of 2.5% NaOCl when they were used in combination.

There is limited knowledge in the literature about the interaction of peracetic acid with NaOCl that reduces the desirable effects of NaOCl (Arias-Moliz et al. 2015). In the present study, there was no statistically significant difference between the single application of NaOCl and its combined use with PAA in terms of eliminating *E. faecalis* biofilms. However, when both solutions are used together, the percentage of dead bacteria is higher. According to the results of the present study, PAA can also be considered as a promising chelating agent like HEBP, thanks to its adequate antibacterial activity when used alone. In addition, it seems that the use of PAA does not reduce the useful properties of NaOCl when two irrigants are used together. However, the findings of other research evaluating different properties of PAA such as smear layer removal capacity, biocompatibility should be considered prior to recommend its use for root canal irrigation.

In the present study, CLSM was used to detect the dead and live bacteria in the experimentally developed biofilms. CLSM was suggested to be an effective method for the identification and measurement of viability without disturbing the attached cells (Chávez et al. 2010; Shen et al. 2010). It is also indicated as a suitable tool for understanding the viability profile and spatial distribution in the biofilms (Del Carpio-Perochena et al. 2011; Zaura-Arite et al. 2001). Therefore, studies evaluating the effect of antibiofilm capacity of antibacterial agents in the root canal prefer to use CLSM for three-dimensional imaging of the bacteria (Rodrigues et al. 2018; Fiallos et al. 2020).

In the present study, *the E.faecalis* biofilm model was experimentally developed to investigate the efficacy of irrigation protocols in removing this microbial flora from the root canal system. However, actual endodontic infections are generally resulted from polymicrobial sources, and *E. faecalis* can interact with other microorganisms and change their behavior in real clinical situations (Hasheminia et al. 2013). The root canal microorganism profiles in real endodontic diseases may differ from those that are created experimentally, and they could react differently to the irrigation agents tested here. This issue can be evaluated as a limitation of this in-vitro study.

Considering the findings of this study, peracetic acid and etidronic acid can be evaluated as alternative chelation agents to EDTA due to their less interaction with NaOCl, reducing its antibacterial effect. Previous research has revealed adequate chelation capacity of PAA in removing the smear layer from root canal dentinal walls (Viola et al. 2022). In the present study, a single PAA showed a similar antibiofilm effect to NaOCl. Therefore, the use of PAA as a single final irrigant can be recommended for both the elimination of microorganisms and the removal of the smear layer. Further studies are needed under in-vivo conditions using the same irrigation agents to support the results of the present study.

## Conclusion

The antimicrobial capacity of 1–2% peracetic acid is similar to its associated use with 2.5% NaOCl regarding eliminating *E.faecalis* biofilms from root canal walls. Therefore, irrigation of infected root canals with a single use of PAA can achieve an adequate antibacterial effect. Lower concentrations of peracetic acid and etidronic acid can be recommended to remove the *E.faecalis* biofilms from root canals to decrease the irrigation solutions’ potential harmful effects.

## Data Availability

No datasets were generated or analysed during the current study.

## References

[CR3] Arias-Moliz MT, Ordinola-Zapata R, Baca P, Ruiz-Linares M, Ferrer-Luque CM (2014) Antimicrobial activity of a sodium hypochlorite/etidronic acid irrigant solution. J Endod 40(12):1999–2002. 10.1016/j.joen.2014.07.031 25266466 10.1016/j.joen.2014.07.031

[CR2] Arias-Moliz MT, Ordinola-Zapata R, Baca P, Ruiz-Linares M, García García E, Hungaro Duarte MA et al (2015) Antimicrobial activity of chlorhexidine, peracetic acid and sodium hypochlorite/etidronate irrigant solutions against *Enterococcus faecalis* biofilms. Int Endod J 48(12):1188–1193. 10.1111/iej.12424 25515403 10.1111/iej.12424

[CR1] Arias-Moliz MT, Morago A, Ordinola-Zapata R, Ferrer-Luque CM, Ruiz-Linares M, Baca P (2016) Effects of dentin debris on the antimicrobial properties of sodium hypochlorite and etidronic acid. J Endod 42(5):771–775. 10.1016/j.joen.2016.01.021 26951957 10.1016/j.joen.2016.01.021

[CR4] Boutsioukis C, Ariaz-Moliz MT (2022) Present status and future directions - irrigants and irrigation methods. Int Endod J 55(3):588–612. 10.1111/iej.13739 35338652 10.1111/iej.13739 PMC9321999

[CR5] Boutsioukis C, Ariaz-Moliz MT, Chavez de Paz LE (2022) A critical analysis of research methods and experimental models to study irrigants and irrigation systems. Int Endod J 55(2):295–329. 10.1111/iej.13710 35171506 10.1111/iej.13710 PMC9314845

[CR6] Briseño-Marroquín B, Callaway A, Shalamzari NG, Wolf TG (2022) Antibacterial efficacy of peracetic acid in comparison with sodium hypochlorite or chlorhexidine against Enterococcus faecalis and Parvimonas Micra. BMC Oral Health 22(1):119. 10.1186/s12903-022-02148-8 35397605 10.1186/s12903-022-02148-8 PMC8994351

[CR7] de Chávez LE, Bergenholtz G, Svensäter G (2010) The effects of antimicrobials on endodontic biofilm bacteria. J Endod 36(1):70–77. 10.1016/j.joen.2009.09.017 20003938 10.1016/j.joen.2009.09.017

[CR8] De-Deus G, Zehnder M, Reis C, Fidel S, Fidel RAS, Galan J (2008) Longitudinal co-site optical microscopy study on the chelating ability of etidronate and EDTA using a comparative single-tooth model. J Endod 34(1):71–75. 10.1016/j.joen.2007.09.020 18155497 10.1016/j.joen.2007.09.020

[CR9] Del Carpio-Perochena AE, Bramante CM, Duarte MA (2011) Biofilm dissolution and cleaning ability of different irrigant solutions on intraorally infected dentin. J Endod 37:1134–1138. 10.1016/j.joen.2011.04.013 21763908 10.1016/j.joen.2011.04.013

[CR10] Fiallos NM, Cecchin D, de Lima CO, Hirata R Jr, Silva EJNL, Sassone LM (2020) Antimicrobial effectiveness of grape seed extract against Enterococcus faecalis biofilm: A confocal laser scanning microscopy analysis. Aust Endod J 46(2):191–196. 10.1111/aej.12390 31814249 10.1111/aej.12390

[CR11] Flach N, Bottcher DE, Parolo CCF, Firmino LB, Malt M, Lammers ML, Grecca FS (2016) Confocal microscopy evaluation of the effect of irrigants on *Enterococcus faecalis* biofilm: an in vitro study. Scanning 38(1):57–62. 10.1002/sca.21241 26153228 10.1002/sca.21241

[CR12] Girard S, Paque F, Badertscher M, Sener B, Zehnder M (2005) Assessment of a gel-type chelating Preparation containing 1-hydroxyethylidene-1, 1-bisphosphonate. Int Endod J 38(11):810–816. 10.1111/j.1365-2591.2005.01021.x 16218973 10.1111/j.1365-2591.2005.01021.x

[CR13] Hasheminia S, Farhad AR, Saatchi M, Rajabzadeh M (2013) Synergistic antibacterial activity of chlorhexidine and hydrogen peroxide against Enterococcus faecalis. J Oral Sci 55(4):275–280. 10.2334/josnusd.55.275 24351914 10.2334/josnusd.55.275

[CR14] Jhajharia K, Parolia A, Shetty KV, Mehta LK (2015) Biofilm in endodontics: a review. J Int Soc Prev Community Dent 5(1):1–12. 10.4103/2231-0762.151956 25767760 10.4103/2231-0762.151956 PMC4355843

[CR15] Kaya S, Yiğit Özer S, Adigüzel Ö (2011) Evaluation of radicular dentin erosion and smear layer removal capacity of Self-Adjusting file using different concentrations of sodium hypochlorite as an initial irrigant. Oral Surg Oral Med Oral Pathol Oral Radiol Endod 112(4):524–530. 10.1016/j.tripleo.2011.02.039 21664155 10.1016/j.tripleo.2011.02.039

[CR16] Kishen A, Haapasalo M (2010) Biofilm models and methods of biofilm assessment. Endodontic Top 22(1):58–78. 10.1111/j.1601-1546.2012.00285.x

[CR17] Lensing HH, Oei HL (1985) Investigations on the sporicidal and fungicidal activity of disinfectants. Zentralbl Bakteriol Mikrobiol Hyg B 181(6):487–495 3938146

[CR18] Lin J, Shen Y, Haapasalo M (2013) Comparative study of biofilm removal with hand, rotary nickel-titanium, and self-adjusting file instrumentation using a novel in vitro biofilm model. J Endod 39(5):658–663. 10.1016/j.joen.2012.11.012 23611386 10.1016/j.joen.2012.11.012

[CR19] Liu H, Wei X, Ling J, Wang W, Huang X (2010) Biofilm formation capability of *Enterococcus faecalis* cells in starvation phase and its susceptibility to sodium hypochlorite. J Endod 36(4):630–635. 10.1016/j.joen.2009.11.016 20307735 10.1016/j.joen.2009.11.016

[CR20] Lottanti S, Gautschi H, Sener B, Zehnder M (2009) Effects of ethylenediaminetetraacetic, etidronic and peracetic acid irrigation on human root dentine and the smear layer. Int Endod J 42(4):335–343. 10.1111/j.1365-2591.2008.01514.x 19220516 10.1111/j.1365-2591.2008.01514.x

[CR21] Mohammadi Z (2008) Sodium hypochlorite in endodontics: an update review. Int Dent J 58(6):329–341. 10.1111/j.1875-595x.2008.tb00354.x 19145794 10.1111/j.1875-595x.2008.tb00354.x

[CR22] Molander A, Reit C, Dahlen G, Kvist T (1998) Microbiological status of root-filled teeth with apical periodontitis. Int Endod J 31(1):1–7 9823122

[CR23] Nair PN, Henry S, Cano V, Vera J (2005) Microbial status of apical root Canal system of human mandibular first molars with primary apical periodontitis after one-visit endodontic treatment. Oral Surg Oral Med Oral Pathol Oral Radiol Endod 99(2):231–252. 10.1016/j.tripleo.2004.10.005 15660098 10.1016/j.tripleo.2004.10.005

[CR24] Nogo-Živanović D, Kanjevac T, Bjelović L, Ristić V, Tanasković I (2019) The effect of final irrigation with MTAD, QMix, and EDTA on smear layer removal and mineral content of root Canal dentin. Microsc Res Tech 82(6):923–930. 10.1002/jemt.23239 30786090 10.1002/jemt.23239

[CR25] Parolia A, Kumar H, Ramamurthy S, Madheswaran T, Davamani F, Pichika MR et al (2021) Effect of propolis nanoparticles against *Enterococcus faecalis* biofilm in the root Canal. Molecules 26(3):715. 10.3390/molecules26030715 33573147 10.3390/molecules26030715 PMC7866495

[CR26] Ricucci D, Siqueira JF Jr (2010) Biofilms and apical periodontitis: study of prevalence and association with clinical and histopathologic findings. J Endod 36(8):1277–1288. 10.1016/j.joen.2010.04.007 20647081 10.1016/j.joen.2010.04.007

[CR27] Rodrigues CT, de Andrade FB, de Vasconcelos LRSM, Midena RZ, Pereira TC, Kuga MC (2018) Antibacterial properties of silver nanoparticles as a root Canal irrigant against Enterococcus faecalis biofilm and infected dentinal tubules. Int Endod J 51(8):901–911. 10.1111/iej.12904 29397005 10.1111/iej.12904

[CR28] Russell RG, Rogers MJ (1999) Bisphosphonates: from the laboratory to the clinic and back. Bone 25(1):97–106. 10.1016/s8756-3282(99)00116-7 10423031 10.1016/s8756-3282(99)00116-7

[CR29] Sedgley C, Lennan SL, Appelbe OK (2005) Survival of *Enterococcus faecalis* in root canals ex vivo. Int Endod J 38(10):735–742. 10.1111/j.1365-2591.2005.01009.x 16164688 10.1111/j.1365-2591.2005.01009.x

[CR30] Shen Y, Stojicic S, Haapasalo M (2010) Bacterial viability in starved and revitalized biofilms: comparison of viability staining and direct culture. J Endod 36(11):1820–1823. 10.1016/j.joen.2010.08.029 20951294 10.1016/j.joen.2010.08.029

[CR31] Silva PV, Guedes DF, Pécora JD, da Cruz-Filho AM (2012) Time-dependent effects of Chitosan on dentin structures. Braz Dent J 23(4):357–361. 10.1590/s0103-64402012000400008 23207849 10.1590/s0103-64402012000400008

[CR32] Siqueira JF Jr, Roças IN (2008) Clinical implications and microbiology of bacterial persistence after treatment procedures. J Endod 34(11):1291–1301e3. 10.1016/j.joen.2008.07.028 18928835 10.1016/j.joen.2008.07.028

[CR33] Sonja S, Shen Y, Haapasalo M (2013) Effect of the source of biofilm bacteria, level of biofilm maturation, and type of disinfecting agent on the susceptibility of biofilm bacteria to antibacterial agents. J Endod 39(4):473–477. 10.1016/j.joen.2012.11.024 23522539 10.1016/j.joen.2012.11.024

[CR34] Souza MA, Bischoff KF, Rigo BDC, Piuco L, Didoné AVL, Bertol CD et al (2021) Cytotoxicity of different concentrations of glycolic acid and its effects on root dentin microhardness - An in vitro study. Aust Endod J 47(3):423–428. 10.1111/aej.12494 33682987 10.1111/aej.12494

[CR35] Tartari T, Guimaraes BM, Amoras LS, Duarte MAH, Silva e Souza PAR, Bramante CM (2015) Etidronate causes minimal changes in the ability of sodium hypochlorite to dissolve organic matter. Int Endod J 48(4):399–404. 10.1111/iej.12329 24893624 10.1111/iej.12329

[CR36] Tartari T, Wichnieski C, Bachmann L, Jafelicci M Jr, Silva RM, Letra A et al (2018) Effect of the combination of several irrigants on dentine surface properties, adsorption of chlorhexidine and adhesion of microorganisms to dentine. Int Endod J 51(12):1420–1433. 10.1111/iej.12960 29862516 10.1111/iej.12960

[CR37] Ulusoy Öİ, Zeyrek S, Çelik B (2017) Evaluation of smear layer removal and marginal adaptation of root Canal sealer after final irrigation using ethylenediaminetetraacetic, peracetic and etidronic acids with different concentrations. Microsc Res Tech 80(7):687–692. 10.1002/jemt.22851 28190294 10.1002/jemt.22851

[CR38] Villalta-Briones N, Baca P, Bravo M, Solana C, Aguado-Pérez B, Ruiz-Linares M, Arias-Moliz MT (2021) A laboratory study of root Canal and isthmus disinfection in extracted teeth using various activation methods with a mixture of sodium hypochlorite and etidronic acid. Int Endod J 54(2):268–278. 10.1111/iej.13417 32970865 10.1111/iej.13417

[CR39] Viola KS, Coaguila-Llerena H, Rodrigues EM, Santos CS, Chavez-Andrade GM, Magro MG et al (2022) Different formulations of peracetic acid: effects on smear layer removal, dentine erosion, cytotoxicity and antibiofilm activity. J Appl Oral Sci Mar 28:30e20210575. 10.1590/1678-7757-2021-0575 10.1590/1678-7757-2021-0575 PMC8963393 35352771

[CR40] Zaura-Arite E, van Marle J, ten Cate JM (2001) Confocal microscopy study of undisturbed and chlorhexidine-treated dental biofilm. J Dent Res 80(5):1436–1440. 10.1177/00220345010800051001 11437215 10.1177/00220345010800051001

